# The PROSITE database for protein families, domains, and sites

**DOI:** 10.1093/nar/gkaf1188

**Published:** 2025-11-20

**Authors:** Christian J A Sigrist, Béatrice A Cuche, Edouard de Castro, Elisabeth Coudert, Nicole Redaschi, Alan Bridge

**Affiliations:** Swiss-Prot Group, Swiss Institute of Bioinformatics (SIB), Centre Médical Universitaire (CMU), 1 rue Michel Servet, CH-1211Geneva 4, Switzerland; Swiss-Prot Group, Swiss Institute of Bioinformatics (SIB), Centre Médical Universitaire (CMU), 1 rue Michel Servet, CH-1211Geneva 4, Switzerland; Swiss-Prot Group, Swiss Institute of Bioinformatics (SIB), Centre Médical Universitaire (CMU), 1 rue Michel Servet, CH-1211Geneva 4, Switzerland; Swiss-Prot Group, Swiss Institute of Bioinformatics (SIB), Centre Médical Universitaire (CMU), 1 rue Michel Servet, CH-1211Geneva 4, Switzerland; Swiss-Prot Group, Swiss Institute of Bioinformatics (SIB), Centre Médical Universitaire (CMU), 1 rue Michel Servet, CH-1211Geneva 4, Switzerland; Swiss-Prot Group, Swiss Institute of Bioinformatics (SIB), Centre Médical Universitaire (CMU), 1 rue Michel Servet, CH-1211Geneva 4, Switzerland

## Abstract

PROSITE (https://prosite.expasy.org/) is a database of entries documenting protein domains, families, and functional sites, along with the associated patterns and profiles used to identify them. It is complemented by ProRule, a rule collection that enhances the discriminatory power of these profiles and patterns by providing additional information about amino acids critical for function and/or structure. Together, PROSITE motifs and ProRules are used to annotate domains and features in UniProtKB/Swiss-Prot entries. Since the onset of the COVID-19 pandemic, PROSITE has contributed to SARS-CoV-2 research by leveraging existing tools and by developing new profiles and ProRules for SARS-CoV-2 protein domains. A newly developed profile has also uncovered a link between coregulators of two transcription factor families: POU2F and NF-κB. ProRule has been updated to incorporate the ChEBI ontology to describe chemical ligands and the Rhea reference vocabulary for biochemical reaction annotation. Predicted tridimensional (3D) structures from AlphaFold are now regularly used to define domain boundaries during profile construction. ScanProsite has been enhanced to allow users to visualize motif matches on AlphaFold-predicted structures. In addition, the original pfsearch code has been fully rewritten and optimized to make efficient use of modern multi-core processors, with a new heuristic implemented to further improve performance.

## Graphical Abstract

**Graphical Abstract:**
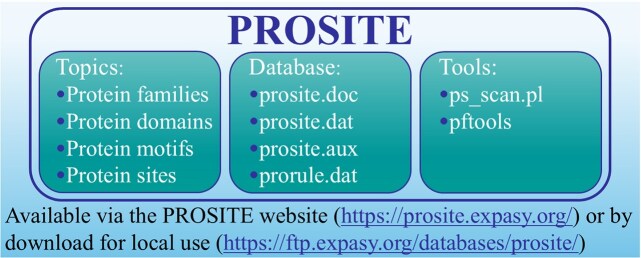

## Introduction

PROSITE is an annotated collection of motif descriptors used to identify protein domains, sites, and families. It is accessible through its web server at https://prosite.expasy.org/ and its components are available for download from https://ftp.expasy.org/databases/prosite/.

When it was first released in 1989, PROSITE contained 58 documentation entries, described by 60 patterns [1]. At the time, the Swiss-Prot database contained around 10 000 entries, and the Protein Data Bank (PDB) included 365 experimentally determined protein structures (according to PDB Statistics). The Translation of EMBL nucleotide sequence database (TrEMBL), which was created only in 1996, did not yet exist. Initially separate resources, Swiss-Prot and TrEMBL were merged in 2003 to form the UniProt Knowledgebase (UniProtKB), serving respectively as its manually reviewed and automatically annotated sections [2].

In 1999, PROSITE was one of the databases involved in creating InterPro: an integrated documentation resource for protein families, domains, and functional sites. InterPro was developed to rationalize the complementary efforts of individual protein signature database projects [3].

Today, PROSITE (release 2025_03 of 18 June 2025) contains 1956 entries, described by 1311 patterns and 1403 profiles, and is supplemented by 1421 ProRules used to annotate UniProtKB/Swiss-Prot entries. In UniProtKB release 2025_03 (18 June 2025), UniProtKB/Swiss-Prot contains 573 661 entries, while UniProtKB/TrEMBL contains 253 061 697 entries [4]. As of the 10 September 2025 release, PDB [5] includes 241 692 experimentally determined protein structures, and the AlphaFold Protein Structure Database [6] provides 214 683 839 predicted structures (July 2025). PROSITE continues to provide new patterns and profiles to InterPro, which has increased the number of its member databases [7].

This paper reviews the evolution of PROSITE over the years to improve the quality of its predictions. It also presents its applications and the updates introduced since our previous publication [8].

## Materials and methods

### Figures

**Figure 1.**
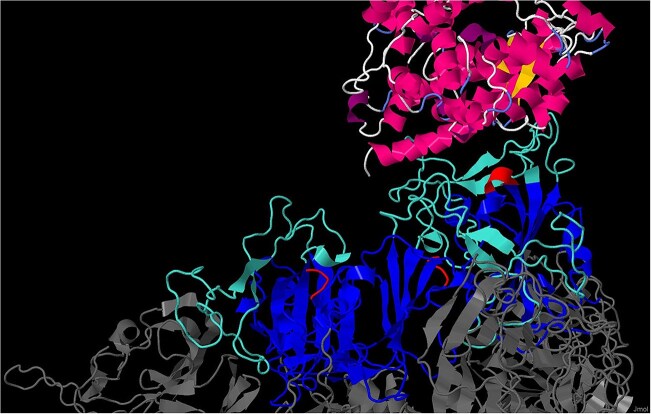
The trimeric SARS-CoV-2 spike protein bound to ACE2. The spike protein is shown in dim grey, the receptor-binding domains (PS51921) in blue, the ACE2-binding regions in turquoise, and the RGD motifs (PS00016) in red. On the top of the figure, the ACE2 protein is shown coloured according to its secondary structures (pink, α-helices; plum, 3_10_-helices; gold, β-strands; light blue, turns).

**Figure 2.**
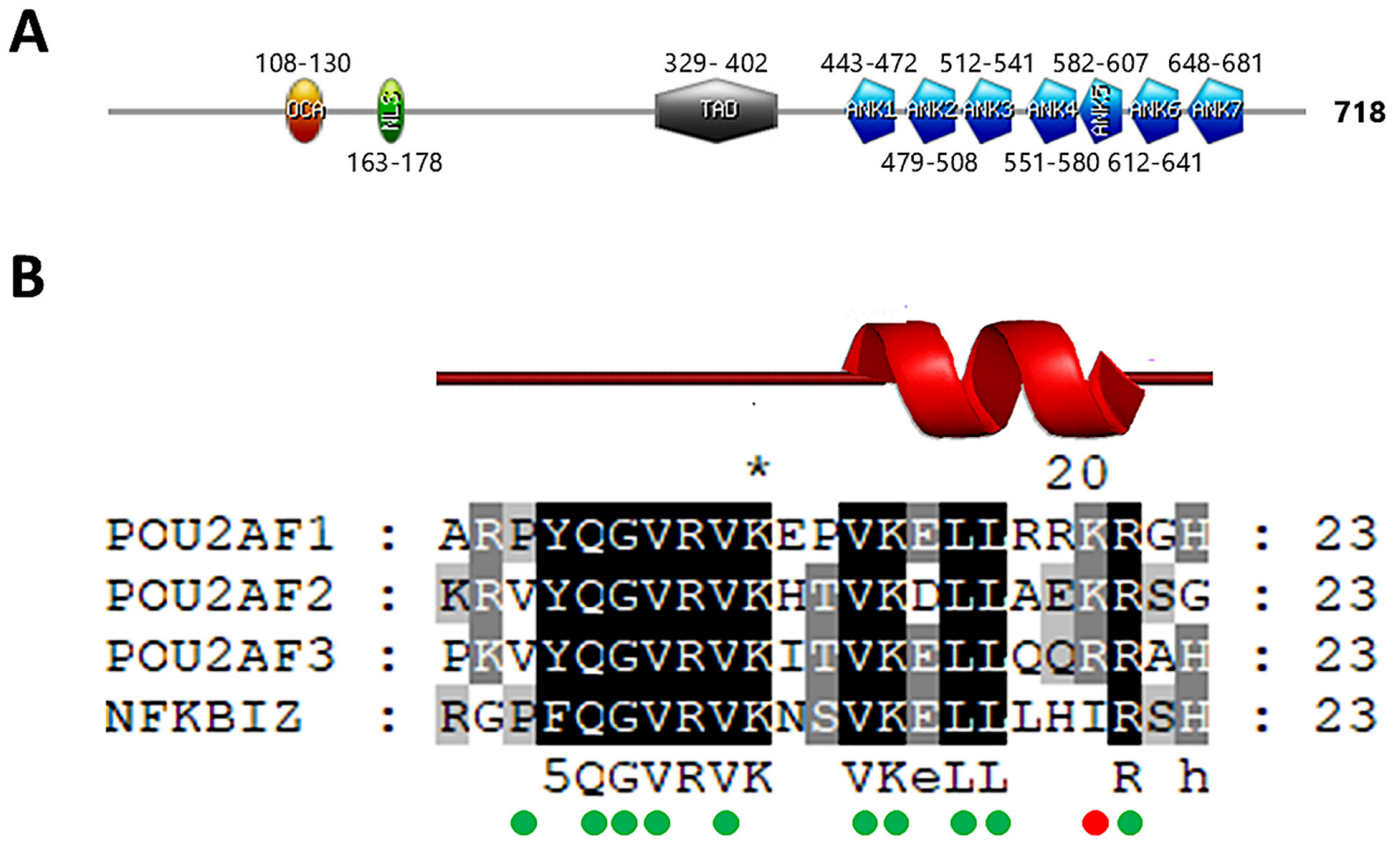
Identification of an OCA motif in IκBζ. (**A**) Domains found in IκBζ: OCA motif (OCA, orange), nuclear localization signal (NLS, green), *trans*-activation domain (TAD, grey), and ankyrin repeats (ANK1-7, blue). (**B**) MSA of the OCA motif of the OCA family members POU2AF1 (OCA-B), POU2AF2 (OCA-T1 or C11orf53), and POU2AF3 (OCA-T2 or COLCA2) and IκBζ. The green circles indicate the conserved residues involved in POU2F1 or DNA binding. The red circle indicates the only residue with a non-conservative substitution in IκBζ. The structure adopted by the OCA motif (PDB: 1CQT) is shown above the alignment.

**Figure 3.**
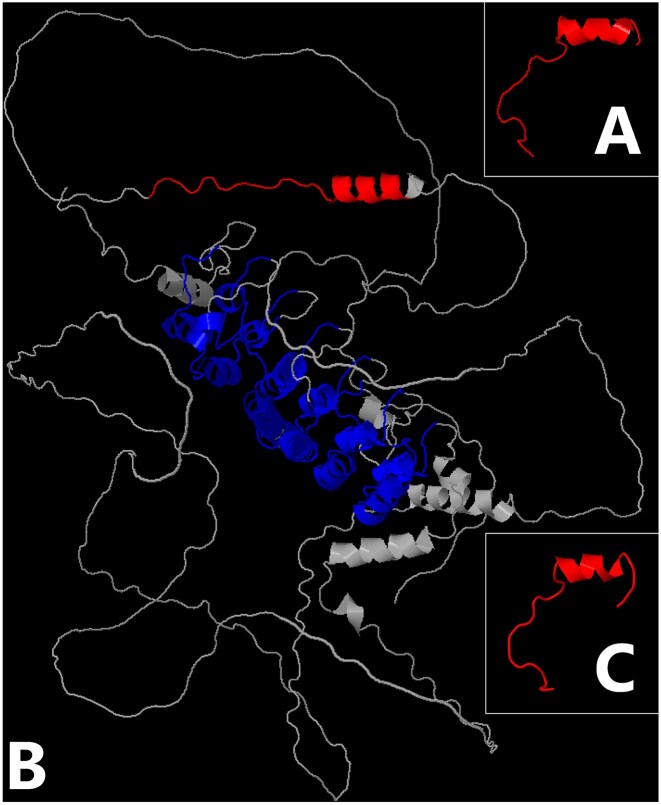
Modelling of the OCA domain. (**A**) The OCA domain of OCA-B (PDB: 1CQT). (**B**) The IκBζ structure predicted by AlphaFold (AF-Q9BYH8-F1-model_v2). The seven ankyrin repeats are represented in blue. (**C**) The human IκBζ OCA domain modelled with SWISS-MODEL using the OCA-B OCA domain (PDB: 1CQT) as a template. The regions shown in red correspond to OCA domains detected by the PROSITE profile.

The 3D structures shown in Figs 1 and 3 were visualized with the JSmol program (https://jmol.sourceforge.net/) used by PROSITE. The secondary structure shown in Fig. 2 was taken from the protein view of 1CQT from PDBsum [9]. The multiple sequence alignment (MSA) shown in Fig. 2 was visualized with the full-featured MSA editor GeneDoc (http://nrbsc.org/gfx/genedoc/).

## The PROSITE database

PROSITE is a database of protein families, domains, and sites. It originally consisted of two files: a documentation file (prosite.doc), which compiles all PROSITE entries describing the families, domains, and sites in the database; and a data file (prosite.dat), which contains PROSITE motifs—either patterns or matrices—designed to identify these elements. The prosite.dat file also included, for each motif, a curated match list for the Swiss-Prot section of UniProtKB [1], as well as an additional match list for PDB since 2003 [10]. In 2004, we introduced a new file, prorule.dat, which contains PROSITE rules (ProRules). These rules notably capture the positions of structurally and/or functionally important amino acids, specify the conditions they must fulfil to play their biological roles, and enable the generation of UniProtKB-formatted annotation for the DE, CC, KW, or FT lines [11, 12].

### prosite.doc

Documentations include a concise description providing useful biological information about protein families, domains, and/or sites [13]. They generally contain all or part of the following information: the origin of the name, the taxonomic occurrence, examples of matching proteins and their domain architectures, the function and, if relevant, the catalytic mechanism, an example of a 3D structure and its description, the main characteristics of the primary sequence, and some references, which often include alignments that helped develop the motif descriptor(s).

### prosite.dat

The motif descriptors used in PROSITE to detect the protein families, domains, or sites described in the documentation are of two types: patterns or profiles [13]. Both are derived from multiple alignments of homologous sequences. This gives these motif descriptors the notable advantage of identifying distant relationships between sequences that would have gone unnoticed based solely on a pairwise sequence alignment. Both patterns and profiles have their own strengths and weaknesses, which define their area of optimum application [13].

#### Patterns

Originally, PROSITE used patterns—also known as regular expressions—as its first motif descriptors [1]. These motifs, typically spanning 10 to 20 amino acids, often correspond to critical functional regions such as enzyme catalytic sites, prosthetic group attachment sites (e.g. haem, pyridoxal-phosphate, biotin), metal ion binding residues, cysteines forming disulphide bonds, and regions involved in binding molecules (e.g. ADP/ATP, GDP/GTP, calcium, DNA) or other proteins. While regular expressions may seem somewhat ‘old-fashioned’ today, they remain widely used by the PROSITE community. Users can scan sequences using either existing patterns from the PROSITE database or custom-defined ones. Moreover, regular expressions continue to be frequently used in scientific publications to describe short, conserved sequence regions of interest. Although the syntax of these patterns may vary somewhat from author to author, they can easily be modified using PROSITE’s own syntax [13].

Some PROSITE motif descriptors are too short and/or degenerate to carry biological significance on their own, as they appear in most known protein sequences. They are marked with the /SKIP-FLAG = TRUE qualifier and are not associated with a match list (see below). By default, they are excluded from ScanProsite analyses; their exclusion needs to be manually deselected. These motifs, some of which predict post-translational modification sites—e.g. N-glycosylation sites, phosphorylation sites, or phosphopantetheine attachment sites—produce matches that are only indicative of a possible function. Independent biological evidence must be considered to confirm the appropriateness of these matches and scans should only be performed against small sets of proteins potentially concerned. Nevertheless, if used appropriately, these patterns can provide very useful information where other methods fail. For example, the N-glycosylation site pattern (https://purl.expasy.org/prosite/signature/PS00001) is used to annotate N-linked (GlcNAc…) asparagines in the extracellular regions of eukaryotic proteins in the UniProtKB/Swiss-Prot database. The accuracy of this annotation process is illustrated by its ability to detect N-glycosylation sites in mammalian interferon-β (IFN-β) that can carry one to five predicted glycosylated asparagines [14]. The pattern correctly predicted the unique human IFN-β N-glycosylation site as well as the three murine sites, all of which were indeed shown to carry N-linked sugars at the predicted positions [14, 15].

Another illustration of the usefulness of such patterns was provided during the COVID-19 pandemic. One of them enabled the identification of an RGD motif (or cell attachment sequence) (https://purl.expasy.org/prosite/signature/PS00016) in the SARS-CoV-2 spike protein (see Fig. 1) [16]. The presence of this motif, potentially capable of interacting with integrins, had gone unnoticed during the initial SARS-CoV-2 genome analysis [17]. Experimental confirmation subsequently showed that this motif, located close to the region involved in angiotensin-converting enzyme 2 (ACE2) binding, can indeed bind integrins [18, 19].

#### Generalized profiles

Sensitive position-specific scoring matrix (PSSM) serves as a very useful scoring matrix that contains evolutionary information of protein sequences, which is commonly used to detect distantly related proteins and protein folding patterns [20]. In 1994, PROSITE introduced an extension of PSSMs known as generalized profiles or weight matrices as motif descriptors [21, 22]. Generalized profiles offer the advantage of being less conservative and covering a larger region, enabling models to be built that cover entire proteins or domains. Their enhanced sensitivity enables poorly conserved domains or proteins to be detected.

Over the past few years, we have continued to create new generalized profiles covering structural domains, which are used for annotating UniProtKB/Swiss-Prot entries and for analysing protein sequences for PROSITE users. Some of them have identified domains in new protein families, revealing unexpected relationships between them. Hence, in 2022, we identified a previously unnoticed link between two transcription factor (TF) families, namely POU2F and nuclear factor (NF)-κB (https://purl.expasy.org/prosite/documentation/PDOC52003). The POU2AF (POU2AF1-3) coactivator proteins have been shown to contain an OCA motif, which forms a ternary complex with an octameric DNA sequence and the POU domain of POU2F (POU2F1-3) TFs to control expression of target genes [23, 24]. A generalized profile built from the OCA motif of POU2AF1 detected the presence of the motif with a significant score not only in the paralogous POU2AF2 and POU2AF3 proteins as expected, but also in NF-κB inhibitor ζ (IκBζ) proteins (Fig. 2A) [25]. Reciprocal searches starting from an IκBζ OCA motif produced a similar result: besides IκBζ OCA motifs, the profile retrieved members of the POU2AF family. Finally, before making our model public and using it to annotate the presence of an OCA motif in IκBζ UniProtKB/Swiss-Prot entries, we made some structural comparisons to strengthen our observations. The OCA motif structure of POU2AF1 has been solved and revealed an N-terminal extended polypeptide strand and a C-terminal two-turn α-helix (Figs 2B and 3A) [24]. Although there is no solved structure for IκBζ, AlphaFold [26] predictions show a large N-terminal globally unstructured region, with few secondary structures, sandwiching the C-terminal domain made of ANK repeats (Fig. 3B). Interestingly, the region mapped by the OCA motif profile on the predicted IκBζ structure is located precisely in one of the few secondary structures present in the largely unstructured N-terminal half and shows a structure like the one observed in POU2AF1, namely an unstructured region followed by an α-helix (Fig. 3). Furthermore, the prediction of the start of the α-helix in IκBζ corresponds exactly to the beginning of the α-helix of the OCA motif in the solved structure (Fig. 2B). However, the N-terminal region preceding the α-helix adopts an elongated structure in the IκBζ structure predicted by AlphaFold as compared to the bends, allowing the corresponding region of POU2AF1 to fit in the major groove of the octamer DNA (Fig. 3). To see if the IκBζ OCA motif could adopt a similar structure, SWISS-MODEL [27] has been used to build an IκBζ OCA motif model using the POU2AF1 OCA motif (PDB: 1CQT) as a template [5, 24]. The modelled structure showed that the IκBζ OCA motif N-terminal region can adopt bends, which should position the conserved residues for interacting both with the octamer DNA major groove and the POU domain (Fig. 3). Among the residues known to interact with the DNA and/or the POU domain of POU2F1, there is only one that is not conserved in the OCA motif of IκBζ, namely the positively charged Lys or Arg at position 20 that makes a hydrogen bond with the POU domain, which is replaced by a hydrophobic aliphatic Ile in IκBζ (Fig. 2B). This substitution would prevent the formation of the hydrogen bond at this position but also extend the hydrophobic surface on the face of the α-helix that binds to a complementary hydrophobic pocket in the POU domain [24]. This increase in the hydrophobic surface could compensate for the loss of the hydrogen bond. As both the primary and secondary structural evidence indicated the presence of a *bona fide* OCA motif in IκBζ, we validated the model. This was made publicly available in PROSITE release 2022_04 on 12 October 2022 and was used to annotate the presence of an OCA motif in the UniProtKB/Swiss-Prot IκBζ entries with the same release date. A year and a half after the OCA motif profile was made publicly available in PROSITE and one year after its integration into InterPro (IPR047571; Release 93.0, 2 March 2023) [7], a paper was published confirming our observations with experimental data [28]. To date, PROSITE remains the only protein domain database to provide a model for the OCA motif.

### prorule.dat

The ProRule database contains a set of manually created rules that provide additional biologically meaningful information about domains detected by PROSITE profiles [11, 12]. Its purpose is to provide domain-specific information in the UniProtKB/Swiss-Prot format, using standardized nomenclature and controlled vocabularies. Occasionally, rules make use of patterns. In these cases, the rules do not work independently but are called by another rule triggered by a profile. ProRule uses the UniRule format [29], which is used for all types of rules created to annotate the UniProtKB, including the HAMAP rules [30].

**Figure 4.**
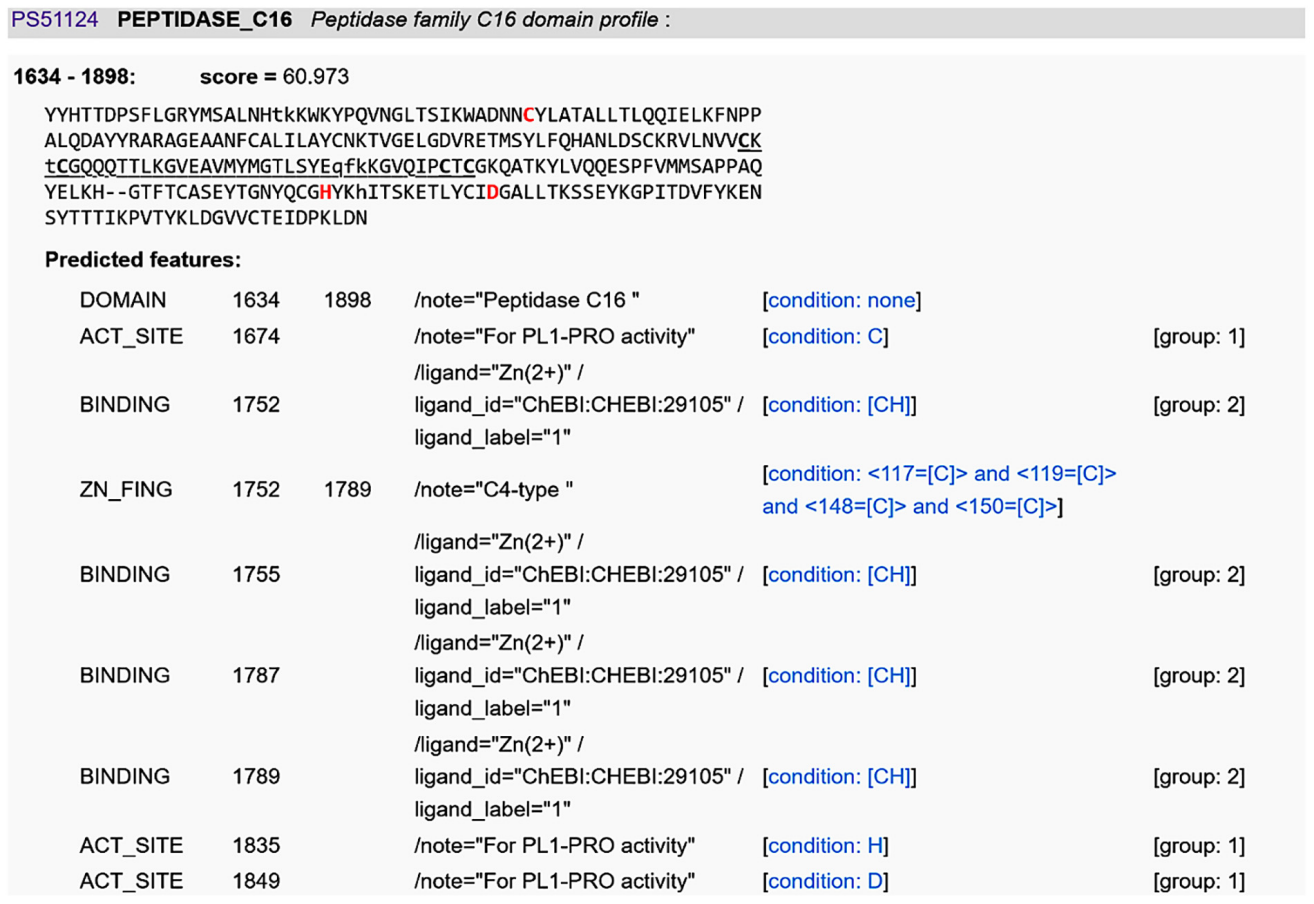
The matched peptidase family C16 [or papain-like (PL) protease] domain in SARS-CoV-2 replicase polyprotein 1ab (P0DTD1; R1AB_SARS2). The catalytic triads C1674–H1835–D1849 and the four zinc-binding C residues of the C4-type zinc finger are detected by the PROSITE profile (PS51124; PEPTIDASE_C16) and its associated ProRule (PRU00444) and are shown in a table on the ScanProsite Results page. The Zn^2+^ ligand is annotated with its unique ChEBI identifier.

ProRule is extensively used by UniProtKB/Swiss-Prot curators to facilitate the annotation work and to check the consistency of UniProtKB/Swiss-Prot entries. Some features of ProRule, like predicted active and binding sites, posttranslationally modified residues (PTMs), or disulphide bonds, are accessible for external users through our ScanProsite web page (Fig. 4) [31].

Like UniProtKB, ProRule now uses the Chemical Entities of Biological Interest (ChEBI) ontology [32] as its reference vocabulary for annotating biologically relevant ligands and their binding sites [33]. Some ProRules, particularly those describing Ca^2+^ and Zn^2+^ binding, have been revised and used to add and/or correct the annotation of residues involved in this binding in Swiss-Prot entries on a large scale, using the ChEBI identifiers. PROSITE users can also benefit from ligand binding site annotations using stable, unique ChEBI identifiers. If ligand binding sites are detected with ScanProsite, they will appear in the results with stable unique identifiers from the ChEBI ontology (Fig. 4). ProRules can also predict potential biochemical reactions performed by a domain, provided that the active site residues are present. These reactions are described using the same reference vocabulary as UniProtKB: Rhea, an expert-curated knowledgebase of biochemical reactions which uses ChEBI to represent reaction participants [34]. However, it should be noted that these predicted reactions should be treated with caution, as profiles can detect evolutionarily distant sequences in different species, and the function may have changed during evolution. Slight differences in reactant binding may result in a related but distinct reaction corresponding to a different Rhea entry [35].

### prosite.aux

Since release 2024_03 (29 May 2024), the file prosite.dat includes only core PROSITE motif data (i.e. patterns and generalized profiles). Auxiliary information—recalculated at each release based on PROSITE motif matches in UniProtKB/Swiss-Prot and PDB—is now distributed in a separate file: prosite.aux.

This file contains, for each motif not flagged as high probability of occurrence (i.e. those lacking the line CC /SKIP-FLAG = TRUE in prosite.dat—see above), the following information:

- Name and accession (ID and AC lines)
- Numerical Results (NR lines): include the UniProtKB release number, the number of UniProtKB/Swiss-Prot entries in that release, and a summary of relevant motif matches
- Taxonomic range comment (CC /TAXO_RANGE line)
- Cross-references to UniProtKB/Swiss-Prot (DR lines), with a curation-assigned status to each sequence:
  - T – true positive (matched and biologically relevant)
  - P – partial sequence (not matched; assumed to be a true positive if the sequence were complete)
  - N – false negative (not matched but expected to match)
  - ? – unknown (matched; biological relevance unclear)
  - F – false positive (matched but not biologically relevant)
- Cross-references to PDB structures (3D lines)

Separating auxiliary information from prosite.dat improves manageability and contributes to more efficient scanning. The auxiliary data—such as DR and 3D lines—plays no role in the scanning process, as it is ancillary to the motifs (patterns and profiles) themselves. By omitting large match lists from the core motif file, memory usage during scanning is reduced. Moreover, updates to auxiliary data no longer require recompilation of the PROSITE data files, making maintenance easier. Finally, pftools imposes a technical limit on the size of the input motif file, which was another key reason for moving DR and 3D lines to the separate prosite.aux file. This reorganization results in a leaner motif file and may lead to a slight increase in scan speed.

## PROSITE availability

### PROSITE web interface

PROSITE can be accessed through a web interface at https://prosite.expasy.org/. The homepage allows access to documentations and motif descriptors (for ProRule there are dedicated pages that can be accessed through a tab) and basic scans of a few sequences (max. 10). More sophisticated scans can be performed with the ScanProsite tool accessible through the dedicated tab. ScanProsite notably allows users to upload their own protein database (max. 16 MB) and to perform scans against protein database with PROSITE motifs or their own pattern [8].

#### Persistent URLs

Each PROSITE documentation entry, motif, and ProRule is assigned a Persistent URL (PURL) via https://purl.archive.org/ [36]. PURLs should be used when referencing specific PROSITE documentations, motifs, or ProRules in publications or data records. A PURL is a stable identifier in the form of a URL that redirects through a resolver to the current location of the resource. This ensures that references remain valid even if the actual URL changes over time.

For example:

- The documentation PDOC52003 can be referenced using: https://purl.expasy.org/prosite/documentation/PDOC52003
- The motif PS52003 uses: https://purl.expasy.org/prosite/signature/PS52003
- The ProRule PRU00444 is accessed via: https://purl.expasy.org/prosite/rule/PRU00444

#### ScanProsite versus UniProtKB/TrEMBL

Redundant sequences do not significantly add to the information content. Their presence slows down and compromises the output of our services, and they are costly to process and perform repetitive computations on. Since the end of 2021, we have restricted scans against the UniProtKB/TrEMBL database to its reference proteomes, i.e. one representative from each cluster of proteomes that are grouped by their overall sequence similarity [4]. Scans using UniProtKB identifiers (IDs) or accession numbers (ACs) are also limited to UniProtKB/Swiss-Prot and reference proteomes entries. However, scans against sequences not belonging to this group are permitted, provided they are supplied in FASTA format.

#### AlphaFold

Since domains correspond to protein units that fold independently, structural information is essential for defining domain boundaries. For many years, PROSITE has used the PDB database [5] to build more precise models with correct domain boundaries. ScanProsite also allows scans against the PDB database and enables visualization of matches on protein structures. For UniProtKB entries associated with a PDB entry, a link to the associated structure is available on the results page, enabling users to view the match on the structure [10]. Unfortunately, PDB, which relies on experimental data, only contains structural information for a limited part of the protein universe. The AlphaFold Protein Structure Database (AlphaFold DB), however, is a vast digital library of predicted protein structures containing over 214 million entries, which greatly increases the size of available structures [6]. AlphaFold uses deep learning models trained on evolutionary relationships and physical constraints to predict a protein’s 3D structure from its amino acid sequence [26]. Given the high quality of these predicted models, we use them to define the boundaries of our profiles. If a precalculated AlphaFold model exists for a UniProtKB entry, ScanProsite now offers the option of visualizing PROSITE hits on the predicted structure. This enables users to evaluate the quality of PROSITE hits in two ways. Firstly, the hit mapped on the AlphaFold predicted structure should resemble the expected domain structure as described in the corresponding PROSITE documentation and PDB match list. If it is completely different, the match is most probably a false positive. Second, partial similarity could indicate an incorrect boundary assessment, and this can be adjusted according to the predicted structure.

### PROSITE for download

The files making up the PROSITE database (prosite.doc, prosite.dat, prosite.aux, and prorule.dat) as well as files containing additional information can be downloaded at https://ftp.expasy.org/databases/prosite/. The tools (ps_scan.pl, pfsearch, pfscan, psa2msa) needed to use them are also available at the same URL in the ps_scan folder as tarballs containing precompiled versions of the tools for various operating systems.

A suite of tools to build and search generalized profiles can be accessed at the following URLs: https://github.com/sib-swiss/pftools3 or https://doi.org/10.5281/zenodo.17360684 [37]. It contains both the original FORTRAN 77 pftools (release 2.3) and the new programme: pftoolsV3. pfsearch2.3 is considered as the standard for PROSITE match lists but, as announced in our previous paper [8], there is now a new version, pfsearchV3, that has been rewritten and optimized to efficiently use modern multi-core processors, and a heuristic has been implemented for further speed enhancements [38]. Except for circular profiles, which require the standard programme pfsearch2.3, most profiles are compatible with both versions of pfsearch. When used with pfsearchV3, circular profiles do not cause the program to crash, but they do not circularize anymore and therefore produce matches of a single unit. ps_scan.pl, PROSITE’s standard analysis tool for patterns and profiles [39], has been adapted to accept both pfsearch2.3 and pfsearchV3. For large-scale scans, we recommend that users who have installed PROSITE locally use pfsearchV3 to reduce the time required. For a limited number of sequences, it is preferable to use pfsearch2.3 to obtain the same reference results as PROSITE and to have the circular profile functionality enabled.

## Conclusion

Since our last article for the NAR database issue [8], PROSITE has continued to develop useful profiles for Swiss-Prot annotation and external users. During the COVID-19 shutdown, we focused particularly on the protein domains present in the SARS-CoV-2 virus in order to offer researchers broad coverage in terms of modules that truly correspond to structural and functional units. An old PROSITE pattern has also proven useful for identifying a new mode of interaction between the virus and the cell surface [16].

Among the new profiles developed, one of them made it possible to identify a previously unidentified link between the POU2F and NF-κB families of TFs. The profile developed for the OCA motif identified in the POU2AF1-3 coactivators of POU2F revealed that the OCA motif was also present in IκBζ, an inhibitor of the NF-κB TF. This discovery allowed PROSITE to annotate the presence of an OCA motif in UniProtKB/Swiss-Prot IκBζ entries more than a year before its presence in these proteins was confirmed experimentally [28].

Thanks to the close collaboration between UniProtKB/Swiss-Prot and PROSITE annotators, the development of new profiles should also be fruitful and lead to new discoveries in the future. To meet the needs of UniProtKB/Swiss-Prot annotators, the ProRules associated with PROSITE profiles and patterns have adopted the ChEBI ontology and the Rhea reference vocabulary to ensure consistent annotation of UniProtKB/Swiss-Prot entries [33].

The newly developed PROSITE motifs continue to be integrated into InterPro [7], of which PROSITE is one of the founding members, and the PROSITE documentation is also largely used for InterPro entry descriptions.

pfsearch2.3 is still the PROSITE reference tool for scanning profiles, but a faster version has been developed, pfsearchV3, which is used in particular for PROSITE scans performed with InterProScan. Both programmes, as well as all the tools needed to install PROSITE locally, are available on our FTP site. The matchlists for UniProtKB/Swiss-Prot and PDB, previously stored with the patterns and profiles in prosite.dat, are now stored separately in a new file, prosite.aux.

Some new features have also been implemented on our website. Our documentation entries, motifs, and ProRules have been supplied with a PURL to ensure that references remain valid even after actual URL changes. When the primary structure of a PDB entry was matched by a PROSITE motif, it was already possible to visualize the match on the tertiary structure. As AlphaFold offers pre-calculated 3D structure predictions for most UniProtKB entries, the output of ScanProsite has been modified to allow users to visualize profile and pattern matches on these 3D models as well.

## Data Availability

PROSITE is copyrighted by the Swiss Institute of Bioinformatics (SIB) and distributed under the Creative Commons Attribution-NonCommercial-NoDerivatives (CC BY-NC-ND 4.0) License. All data are available from the main website (https://prosite.expasy.org/) and can be downloaded from the FTP site (https://ftp.expasy.org/databases/prosite/). The ScanProsite tool can be accessed programmatically through a RESTful web service. Clients send HTTP GET or POST requests, and results are returned directly as plain data in formats such as txt, xml and json. The endpoint is https://prosite.expasy.org/cgi-bin/prosite/scanprosite/PSScan.cgi. For details see https://prosite.expasy.org/scanprosite/scanprosite_doc.html#rest. The pftools are available at https://github.com/sib-swiss/pftools3 or https://doi.org/10.5281/zenodo.17360684.
